# Effect of probenecid on the whole-body disposition of 6-bromo-7-[^11^C]methylpurine in humans assessed with long axial field-of-view PET/CT

**DOI:** 10.1007/s00259-025-07121-5

**Published:** 2025-02-08

**Authors:** Matthias Jackwerth, Severin Mairinger, Ivo Rausch, Maria Weber, Anselm Jorda, Lukas Nics, Werner Langsteger, Markus Zeitlinger, Marcus Hacker, Oliver Langer

**Affiliations:** 1 Department of Clinical Pharmacology, Medical University of Vienna, Vienna, Austria; 2 Department of Biomedical Imaging and Image-guided Therapy, Medical University of Vienna, Vienna, Austria; 3 QIMP Team, Center for Medical Physics and Biomedical Engineering, Medical University of Vienna, Vienna, Austria

**Keywords:** Long axial field-of-view PET/CT, Membrane transporters, Drug disposition, Transporter-mediated drug-drug interaction, Probenecid

## Abstract

**Purpose:**

Multidrug resistance-associated proteins (MRPs) have a widespread tissue distribution. They play an important role in drug disposition and drug-drug interactions (DDIs) and have been associated with various diseases. PET with 6-bromo-7-[^11^C]methylpurine ([^11^C]BMP) has been used to assess MRP1 function in the brain and lungs of mice. [^11^C]BMP crosses cellular membranes by passive diffusion followed by intracellular conjugation with glutathione and MRP1-mediated efflux of the radiolabelled glutathione-conjugate. In this study, we assessed the effect of the prototypical organic anion transporter inhibitor probenecid on the whole-body disposition of [^11^C]BMP to examine its suitability for measuring the function of MRP1 and possibly other MRP subtypes across multiple tissues.

**Methods:**

Seven healthy volunteers (3 women, 4 men) underwent two dynamic whole-body PET scans on a long axial field-of-view (LAFOV) PET/CT system after intravenous injection of [^11^C]BMP, without and with pre-treatment with a single oral dose of probenecid. Volumes of interest were outlined for several MRP-expressing tissues (cerebral cortex, cerebellum, choroid plexus, retina, lungs, myocardium, skeletal muscle, kidneys, and liver). Tissue time-activity curves were corrected for the contribution of vascular radioactivity and the elimination rate constant (*k*_E_, h^− 1^) was calculated as a parameter for tissue MRP function.

**Results:**

Radioactivity was primarily excreted into the urinary bladder and urinary clearance was significantly decreased after probenecid administration (− 50 ± 16%). Following probenecid administration, *k*_E_ was significantly decreased in the kidneys (− 43 ± 20%), liver (− 18 ± 15%), myocardium (− 16 ± 12%), skeletal muscle (− 51 ± 34%), and retina (− 57 ± 29%, non-blood-corrected).

**Conclusion:**

Our study highlights the great potential of LAFOV PET/CT to assess drug disposition and transporter-mediated DDIs in humans at a whole-body, multi-tissue level. Due to the slow elimination of [^11^C]BMP-derived radioactivity from the human brain, [^11^C]BMP appears unsuitable to measure cerebral MRP1 function in humans, but it may be used to assess the function of MRP1 and possibly other MRP subtypes in various peripheral tissues.

**Trial registration:**

EudraCT 2021-006348-29. Registered 15 December 2021.

**Supplementary Information:**

The online version contains supplementary material available at 10.1007/s00259-025-07121-5.

## Introduction

The adenosine triphosphate-binding cassette (ABC) transporter family comprises 49 individual members in humans [1, 2]. These transporters use the energy of adenosine triphosphate to translocate their substrates across extracellular and intracellular membranes. ABC transporters have a widespread tissue distribution and can transport a broad spectrum of endogenous and exogenous substances including drugs and their metabolites. A number of ABC transporters are abundantly expressed in excretory organs (i.e., kidneys and liver), in the intestine, and at blood-tissue barriers (e.g., blood-brain barrier, BBB), where they mediate the urinary and biliary excretion of drugs and drug metabolites, restrict intestinal absorption, and limit the tissue distribution of drugs [3]. ABC transporters play an important role in pharmacokinetic drug-drug interactions (DDIs) [3]. A DDI can occur when a drug which is a transporter substrate is co-administered with a second drug which inhibits the transporter. This can profoundly alter the plasma and tissue pharmacokinetics of the substrate drug and cause serious adverse effects [3]. Apart from their crucial role in drug disposition, ABC transporters have been associated with various diseases and may for instance contribute to multidrug resistance of tumours [4].

Positron emission tomography (PET) imaging with radiolabelled drugs has been proposed as a tool to assess transporter-mediated DDIs in human tissues [5]. Given the widespread tissue distribution of ABC transporters, an imaging-based DDI assessment would ideally cover the whole body. This would allow simultaneous assessment of drug pharmacokinetics in organs targeted for treatment as well as in organs relevant for drug toxicity. However, due to the limited axial field of view (FOV) of previously available clinical PET scanners, the assessment of drug disposition at a whole-body level has so far only been possible in rodents [6]. The recent availability of clinical PET systems with a long axial FOV (LAFOV) overcomes this limitation allowing dynamic whole-body imaging in humans [7–9].

The PET tracer 6-bromo-7-[^11^C]methylpurine ([^11^C]BMP) has been used to measure the function of multidrug resistance-associated protein 1 (MRP1/ABCC1) in the rodent brain and lungs [10–14]. [^11^C]BMP is a “pro-tracer”, which crosses cellular membranes by passive diffusion followed by intracellular conversion into its glutathione-conjugate *S*-(6-(7-[^11^C]methylpurinyl))glutathione ([^11^C]MPG), which is exported from cells by MRP1 and possibly by other MRP subtypes [10, 12, 15]. In a previous study, we reported test-retest variability (TRTV) data in healthy volunteers, sex differences in tissue MRP function and the human dosimetry of [^11^C]BMP [16].

In this exploratory study, we assessed the suitability of [^11^C]BMP to measure the function of MRP1 and possibly other MRP subtypes across multiple human tissues. To this end, we performed dynamic whole-body PET scans in healthy subjects on a LAFOV PET/CT system [9] after intravenous (i.v.) injection of [^11^C]BMP, under baseline conditions and after administration of the MRP inhibitor probenecid.

## Materials and methods

### General

This exploratory study was conducted in accordance with the ICH-GCP guidelines and the Declaration of Helsinki. The trial was registered in the EudraCT database (2021-006348-29) and was approved by the Ethics Committee of the Medical University of Vienna and the Austrian Agency for Health and Food Safety. All subjects gave oral and written informed consent before enrolment in the study. Seven healthy subjects (4 men: age: 28 ± 2 years, weight: 85 ± 11 kg and 3 women: age: 25 ± 1 years, weight: 68 ± 4 kg) were included into the study. Subjects were free of any medication for at least 14 days and judged as healthy based on clinical examination and routine blood and urine laboratory assessments.

### PET/CT and MR imaging

Each subject underwent two dynamic whole-body PET scans after i.v. injection of [^11^C]BMP on a Biograph Vision Quadra PET/CT system (Siemens Healthineers, Knoxville, TN, USA) (axial FOV: 106 cm) as described previously [16]. The first scan was a baseline scan and the second scan was acquired at approximately 3 h after oral intake of a single dose (2 g) of probenecid (Probenecid Biokanol^®^ 500-mg tablets, Biokanol^®^ Pharma GmbH, Rastatt, Germany). The mean interval between the two scans was 22 ± 43 days. After acquiring a low-dose computed tomography (CT) scan (CareDose4D, CarekV setting: Semi, reference tube voltage: 100 kVp with tin filter, reference tube current: 30 mAs) for attenuation correction, [^11^C]BMP (385 ± 34 MBq, containing < 50 µg of unlabelled BMP), which had been synthesised as described before [17], was administered as an i.v. bolus over 20 s. Simultaneously, a 90-min list mode PET acquisition was started and venous blood samples were collected at 5, 10, 20, 30, 40, 60, and 90 min after radiotracer injection. Radioactivity in blood and plasma aliquots was measured in a gamma counter (Perkin Elmer 1480 Wizard 3 gamma counter, Meriden, CT, USA), which had been cross-calibrated with the PET/CT scanner. Plasma samples obtained at 5, 20, and 40 min after radiotracer injection were analysed with radio-high-performance liquid chromatography (radio-HPLC) to assess the percentages of [^11^C]BMP and [^11^C]MPG as described before [16]. After the imaging session, subjects were asked to empty their urinary bladder. An undiluted 2-mL urine sample was analysed with radio-HPLC to assess the percentage of [^11^C]MPG in urine. On a separate day after the PET/CT examination, a T1-weighted magnetic resonance imaging (MRI) scan of the brain was acquired on a Siemens Magnetom Skyra 3T MR system.

### Data analysis

The PET list mode data were re-binned into 1 × 15 s, 3 × 5 s, 3 × 10 s, 2 × 30 s, 3 × 60 s, 2 × 150 s, 2 × 300 s, and 7 × 300 s frames and each PET frame was reconstructed into a 440 × 440 × 531 matrix (voxel size: 1.65 × 1.65 × 2 mm^3^) with an ordinary Poisson ordered-subset expectation-maximisation algorithm (4 iterations, 5 subsets) with PSF modelling and TOF information (corresponding to standard reconstruction settings for such a system [18]). A 2-mm FWHM Gaussian post-reconstruction filter was applied to all images. Scatter correction along with a CT-attenuation correction was applied to all PET data. Volumes of interest (VOIs) were outlined for the descending aorta, right lung, myocardium, skeletal muscle (right triceps brachii), right kidney cortex, liver, urinary bladder, and gall bladder (including the extrahepatic bile duct) in the PFUS tool in PMOD (version 4.404, PMOD Technologies Ltd., Zürich, Switzerland) as described previously [16]. The brain kinetics of [^11^C]BMP were analysed using a brain region atlas (N30R83) implemented in the PNEURO tool in PMOD. The anatomical MRI was segmented into grey and white matter and matched to a Montreal Neurological Institute (MNI) T1-MRI template before transferring the atlas regions to the PET data. As described in our previous study [16], we selected a global cortical grey matter VOI, composed of all cerebral cortical regions provided by the brain atlas, and a cerebellar grey matter VOI, because the latter showed higher radioactivity concentration than the rest of the brain. The retina, choroid plexus, and ventricles were manually outlined on the MRI data using the PNEURO tool as described previously [16]. From the VOIs, time-activity curves (TACs) in units of standardised uptake value (SUV) were extracted. For the urinary bladder and gall bladder, TACs were expressed in units of percentage of the administered activity. TACs for the cerebral cortex, cerebellum, lungs, myocardium, skeletal muscle, kidneys, and liver were corrected for the vascular contribution of radioactivity by subtraction of the TAC of the descending aorta multiplied by the respective tissue blood volume fraction obtained from literature (brain: 0.052 [19], lungs: 0.16 [20], myocardium: 0.14 [21], skeletal muscle: 0.04 [22], kidney cortex: 0.10 [23], and liver: 0.33 [24]). The retina and choroid plexus TACs could not be corrected for blood radioactivity due to a lack of fractional blood volume data from literature.

For selected MRP-expressing tissues (i.e., cerebral cortex, cerebellum, choroid plexus, retina, lungs, myocardium, skeletal muscle, kidneys, and liver), the elimination rate constant (*k*_E_, h^− 1^) was determined as a parameter for tissue MRP function [16]. *k*_E_ represents the slope of the linear part of the natural logarithm-transformed blood-corrected tissue TACs from 15 to 90 min after radiotracer injection and was obtained by linear regression analysis using Microsoft^®^ Excel^®^ 2019 MSO.

A logarithmic trendline ($\:y=c\times\:ln\left(x\right)+b$) was fitted to the plasma-to-blood ratios of total radioactivity *versus* time (measured in the gamma counter from sampled blood and plasma) and an exponential trendline ($\:y=c\:\times\:\:{e}^{bx}$) was fitted to the HPLC-determined fraction of [^11^C]MPG in plasma *versus* time using Microsoft® Excel®. These were then applied to the image-derived TACs from the descending aorta to obtain the TACs of total radioactivity or the TACs of [^11^C]MPG in arterial plasma for time points ≥ 5 min. The urinary clearances (mL/min) of total radioactivity with respect to the plasma concentration (CL_urine, plasma, total_) or kidney concentration (CL_urine, kidney, total_) were calculated by dividing the amount of total radioactivity (kBq) in the urinary bladder in the last PET frame by the area under the curve (AUC, kBq/mL×min) of total radioactivity in plasma from 5 to 90 min or by the AUC of the blood-corrected kidney TAC, respectively. The urinary clearance of [^11^C]MPG with respect to the plasma concentration (CL_urine, plasma, [_^11^_C]MPG_) was calculated by dividing the amount of [^11^C]MPG in the urinary bladder in the last PET frame (obtained by multiplying the amount of total radioactivity in the urinary bladder with the HPLC-determined fraction of [^11^C]MPG) by the AUC of [^11^C]MPG in plasma from 5 to 90 min.

### Statistical analysis

Because of the exploratory nature of this study no formal sample size calculation and adjustment for multiple comparisons were performed. However, based on the previously determined coefficient of variation of *k*_E_ in the brain and lungs of C57BL/6J mice of 5% [12], 6 subjects would yield 80% power to detect at least a 10% change in *k*_E_ at a significance level of 0.05. Statistical analysis was performed using Prism 10.2.3 (Graphpad Software, Dotmatics, Boston, MA, USA). Outcome parameters for baseline and probenecid scans were compared using a two-sided, paired t-test. All values are expressed as mean ± standard deviation (SD).

## Results

**Fig. 1:**
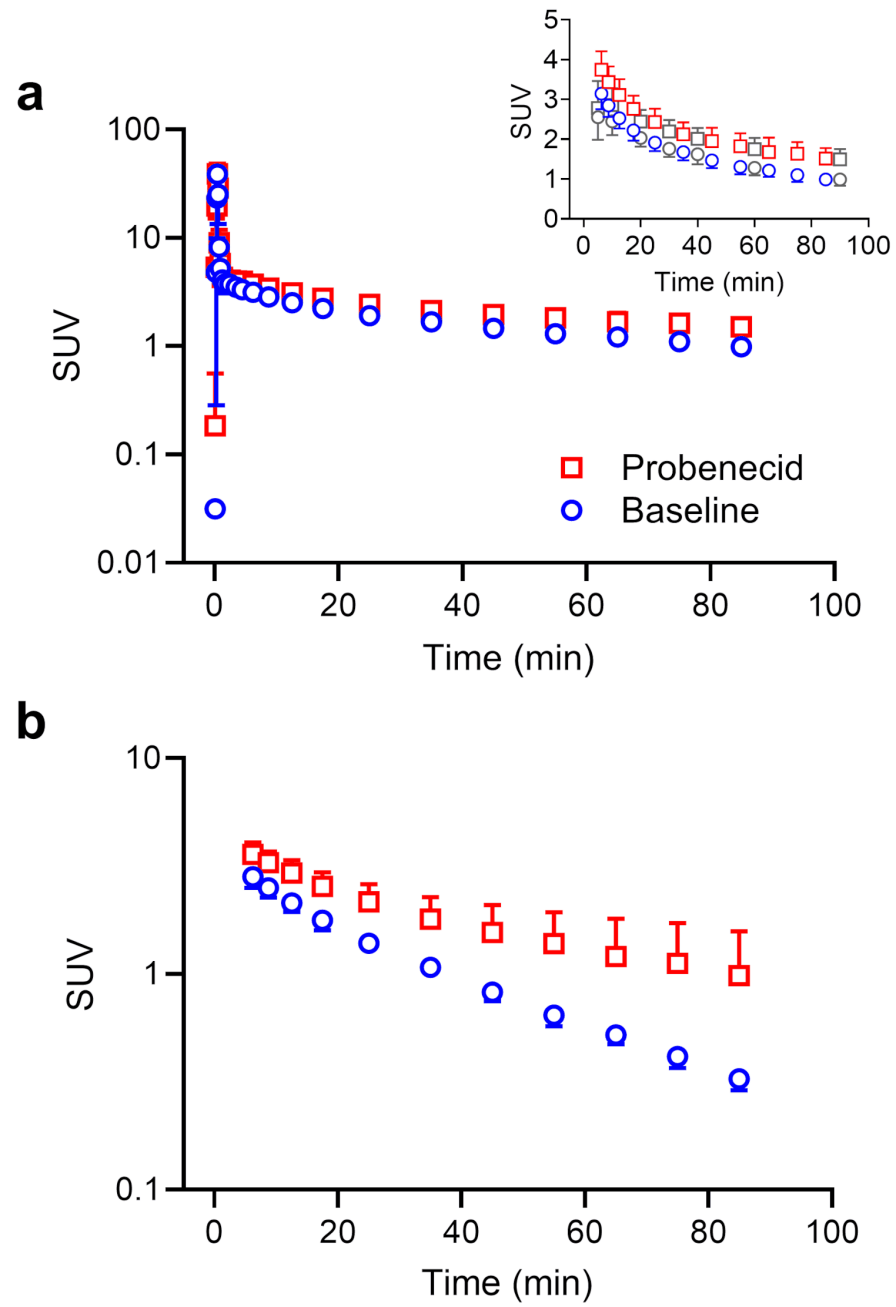
Mean (± SD) time-activity curves of total radioactivity in arterial blood (image-derived from the descending aorta, (**a**) and of the radiolabelled glutathione-conjugate [^11^C]MPG in arterial plasma (**b**) for baseline scans and scans after probenecid administration (*n* = 7). The insert in panel A additionally shows radioactivity concentrations in sampled venous blood measured in a gamma counter (grey symbols)

We included 7 subjects in our study, who underwent two dynamic whole-body PET scans on a LAFOV PET/CT system after i.v. injection of [^11^C]BMP, without and with probenecid pre-treatment. All study-related procedures were well tolerated without any serious adverse events. We generated an image-derived blood curve (Fig. 1a) by placing a VOI into the descending aorta. Image-derived blood measurements agreed well with those from sampled venous blood measured in a gamma counter, for time points ≥ 5 min (Fig. 1a, insert). We analysed venous plasma samples obtained at 5, 20, and 40 min after radiotracer injection with radio-HPLC (Supplementary Fig. 1). At all time points, the major radiolabelled species in plasma was the radiolabelled glutathione-conjugate [^11^C]MPG. In addition, some unconverted [^11^C]BMP was detected and one unidentified radiolabelled species, which eluted after [^11^C]BMP and which percentage increased over time (Supplementary Fig. 1). At all three time points, the percentage of [^11^C]MPG in plasma was significantly higher after probenecid administration than at baseline (5 min, baseline: 66 ± 5%, probenecid: 69 ± 3%, *p* ≤ 0.01; 20 min, baseline: 47 ± 4%, probenecid: 57 ± 11%, *p* ≤ 0.05; 40 min, baseline: 40 ± 3%, probenecid: 54 ± 11%, *p* ≤ 0.05). The elimination rate of [^11^C]MPG from plasma was significantly decreased after probenecid administration (plasma *k*_E_, baseline: 1.482 ± 0.100 h^− 1^, probenecid: 0.910 ± 0.295 h^− 1^, − 38 ± 21%, *p* ≤ 0.01, Fig. 1b).

**Fig. 2:**
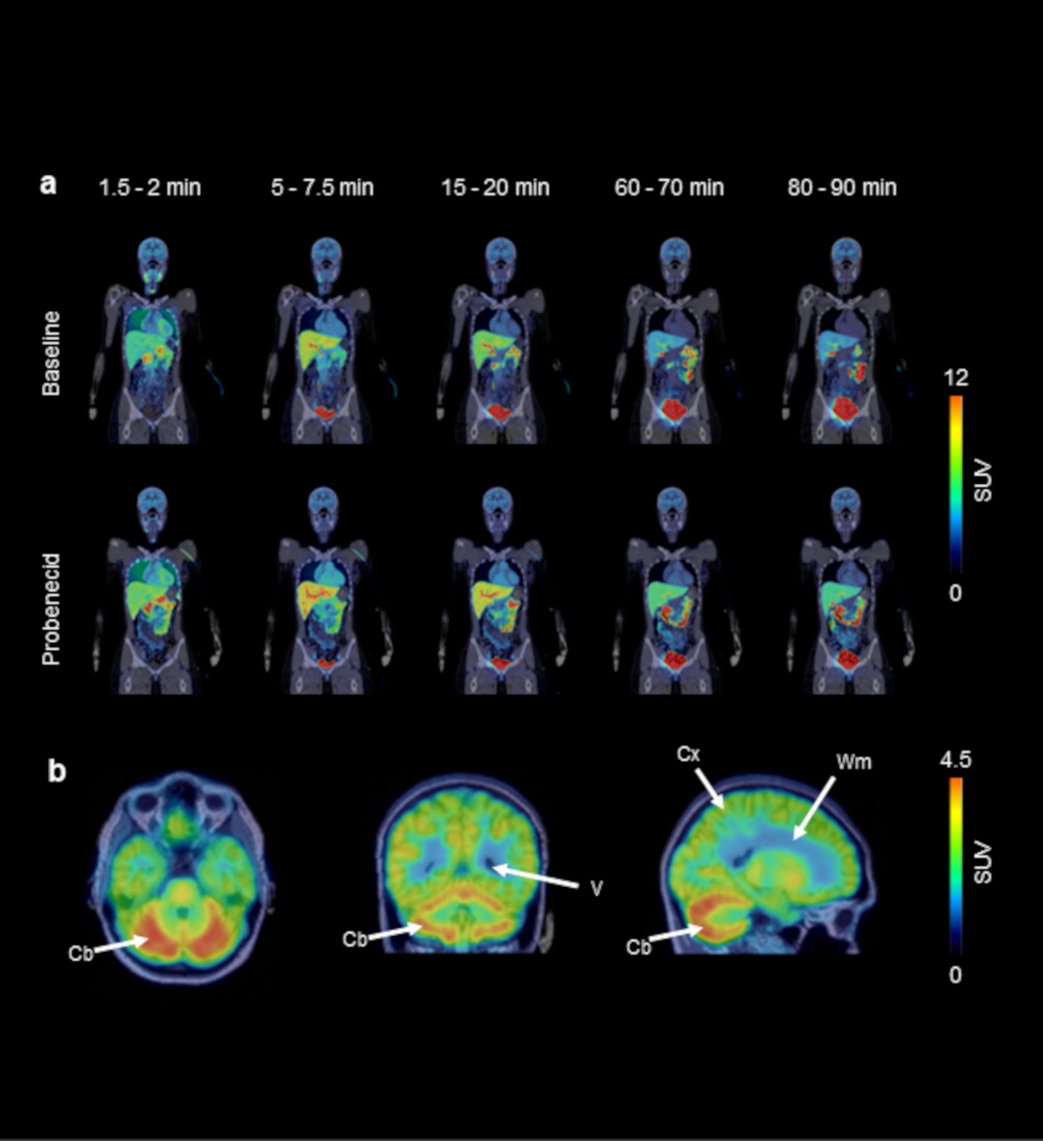
Coronal whole-body PET/CT images obtained at different time points after injection of [^11^C]BMP in one representative female subject for the baseline scan and the scan after probenecid administration (**a**) and MR-co-registered PET average images (0–90 min) of the brain in horizontal, coronal and sagittal planes of the same subject for the baseline scan (**b**). In **b**, anatomical structures are labelled with white arrows (Cb, cerebellum; Cx, cortex; Wm, white matter; V, left lateral ventricle)

**Fig. 3:**
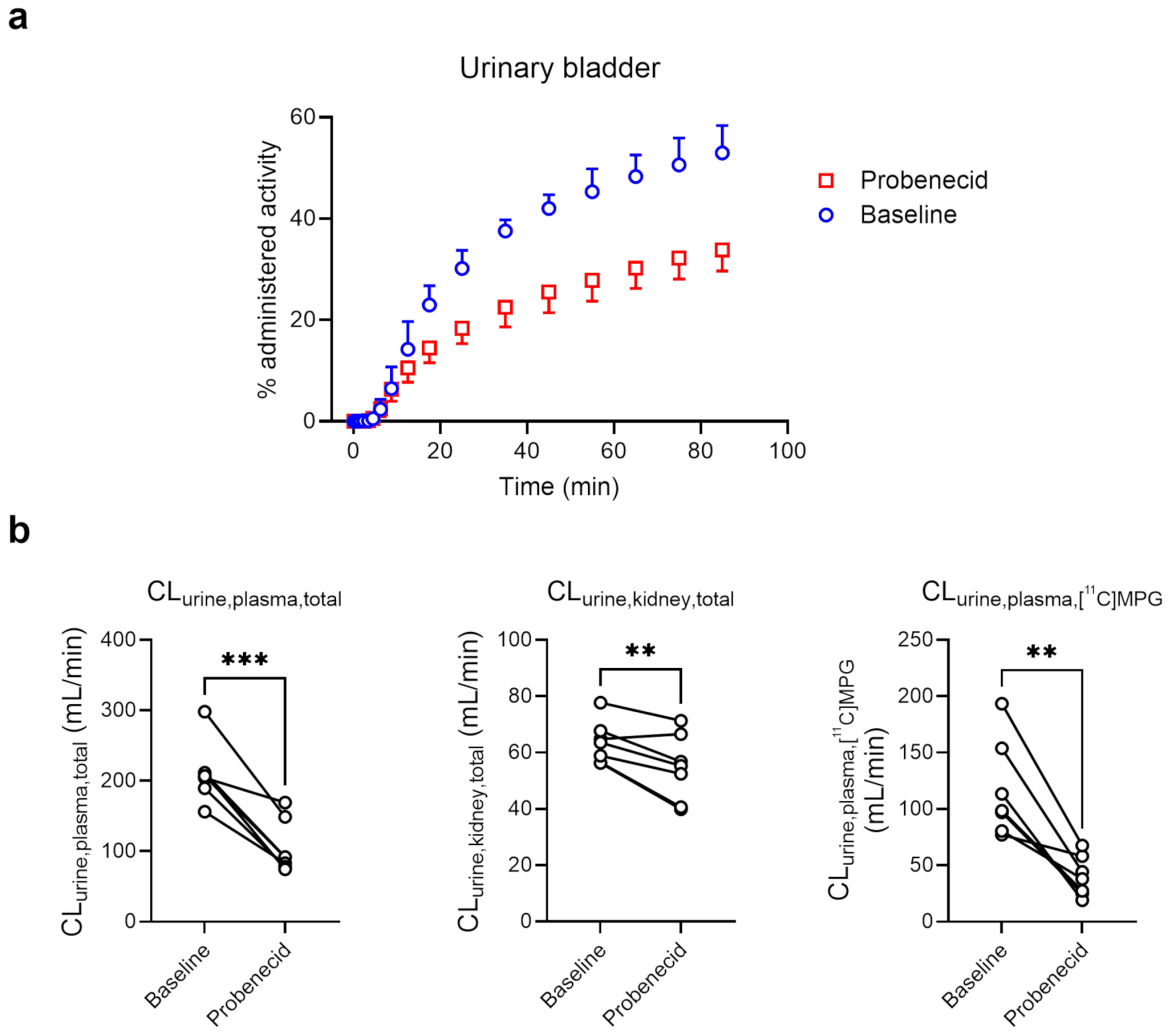
Mean (± SD) time-activity curves for the excretion of radioactivity into the urinary bladder (**a**), urinary clearance of total radioactivity with respect to the plasma concentration (CL_urine, plasma, total_) or kidney concentration (CL_urine, kidney, total_), and urinary clearance of [^11^C]MPG with respect to the plasma concentration (CL_urine, plasma, [_^11^_C]MPG_) (**b**) for baseline scans and scans after probenecid administration (*n* = 7). **, *p* ≤ 0.01; ***, *p* ≤ 0.001; two-sided, paired t-test

Over the time course of the PET scan, radioactivity was predominantly excreted into the urinary bladder. Figure 2a shows representative whole-body PET/CT images without and with probenecid administration and Fig. 3a TACs for the excretion of radioactivity into the urinary bladder. Probenecid administration significantly decreased the amount of activity excreted into the urinary bladder (% administered activity in the urinary bladder in the last PET frame, baseline: 53 ± 5%, probenecid: 34 ± 4%, − 35 ± 15%, *p* ≤ 0.01, Fig. 3a). Radio-HPLC analysis of urine collected at the end of the PET scan (Supplementary Fig. 2) revealed the presence of several radiolabelled species with [^11^C]MPG representing 23 ± 7% of total radioactivity for the baseline and 20 ± 7% for the probenecid scan.

We calculated the urinary clearances of total radioactivity with respect to the plasma (CL_urine, plasma, total_) or kidney (CL_urine, kidney, total_) concentrations, which were both significantly decreased after probenecid administration with greater decreases for CL_urine, plasma, total_ (− 50 ± 16%, *p* ≤ 0.001) than for CL_urine, kidney, total_ (− 15 ± 11%, *p* ≤ 0.01) (Fig. 3b). Also, the urinary clearance of [^11^C]MPG with respect to the plasma concentration (CL_urine, plasma, [_^11^_C]MPG_) was significantly decreased after probenecid administration (− 63 ± 19%, *p* ≤ 0.01) and to a greater extent than CL_urine, plasma, total_ (Fig. 3b). In contrast to the pronounced urinary excretion of radioactivity, biliary excretion was rather low and not significantly different between baseline and probenecid scans (% administered activity in the gall bladder in the last PET frame, baseline: 3 ± 3%, probenecid: 4 ± 3%, Supplementary Fig. 3).

**Fig. 4:**
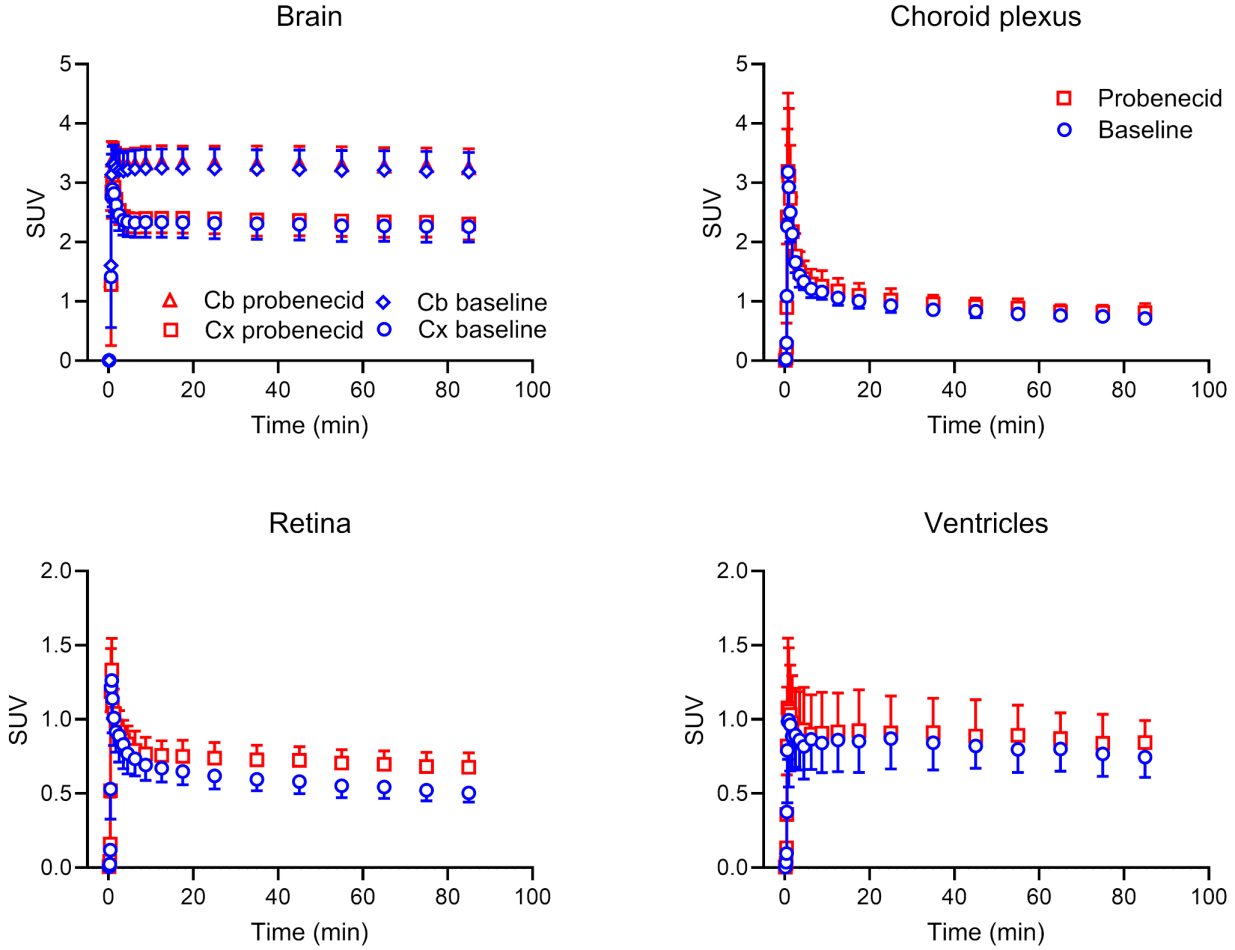
Mean (± SD) time-activity curves in central MRP-expressing tissues, i.e., brain (cortex, Cx and cerebellum, Cb), choroid plexus, and retina, and in the ventricles for baseline scans and scans after probenecid administration (*n* = 7). TACs for the cortex and cerebellum are corrected for the vascular content of radioactivity

**Fig. 5:**
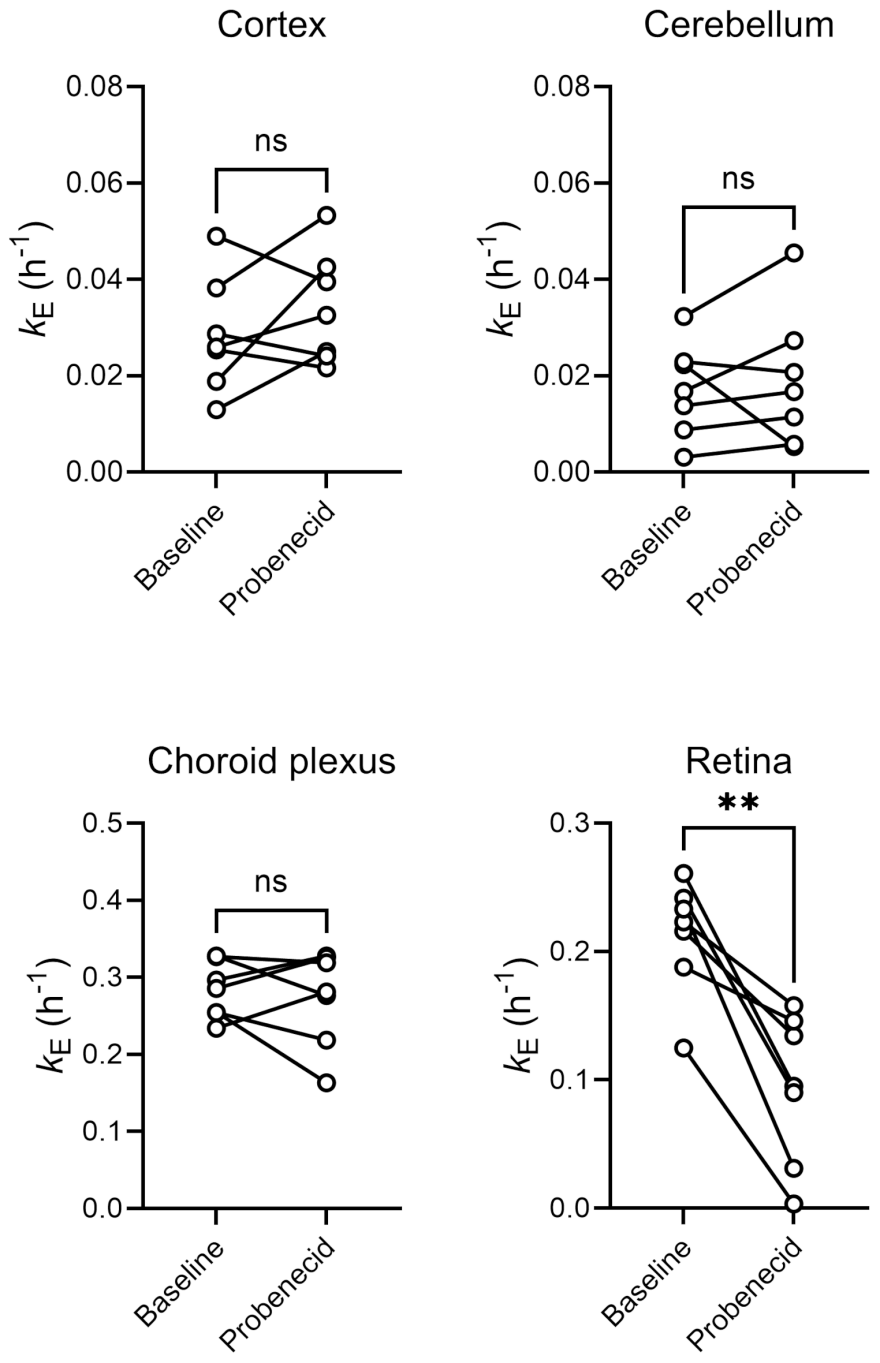
Rate constant for radioactivity elimination (*k*_E_, h^− 1^) from central MRP-expressing tissues, i.e., cortex, cerebellum, choroid plexus, and retina, in individual subjects for baseline scans and scans after probenecid administration (*n* = 7). ns, not significant; **, *p* ≤ 0.01; two-sided, paired t-test

We analysed several MRP-expressing central and peripheral tissues. In Supplementary Figs. 4 and 5, the localisation of MRP1 and other MRP subtypes in the analysed central and peripheral tissues is illustrated. TACs for central tissues (i.e., cortex, cerebellum, choroid plexus, and retina) are shown in Fig. 4 and representative MR-co-registered PET average images of the brain in Fig. 2b. Radioactivity preferentially accumulated in grey matter with a markedly higher uptake (∼ 1.4-fold) in cerebellar than in cortical grey matter (Figs. 2b and 4). As an outcome parameter for tissue MRP function, we calculated *k*_E_ values (Fig. 5, Supplementary Table 1). While the cortex and cerebellum showed a very slow elimination of radioactivity, the choroid plexus and retina showed faster radioactivity elimination (Fig. 5). We additionally analysed radioactivity kinetics in the ventricular system. The shape of the ventricular TACs (Fig. 4) was similar to the brain TACs, but activity levels were 3 to 4 times lower than in the cortex and cerebellum. The retina was the only central tissue, in which *k*_E_ was significantly decreased after probenecid administration (Fig. 5, Supplementary Table 1). The change in *k*_E_ after probenecid administration exceeded the previously determined TRTV of *k*_E_ in this tissue (Supplementary Table 1).

**Fig. 6:**
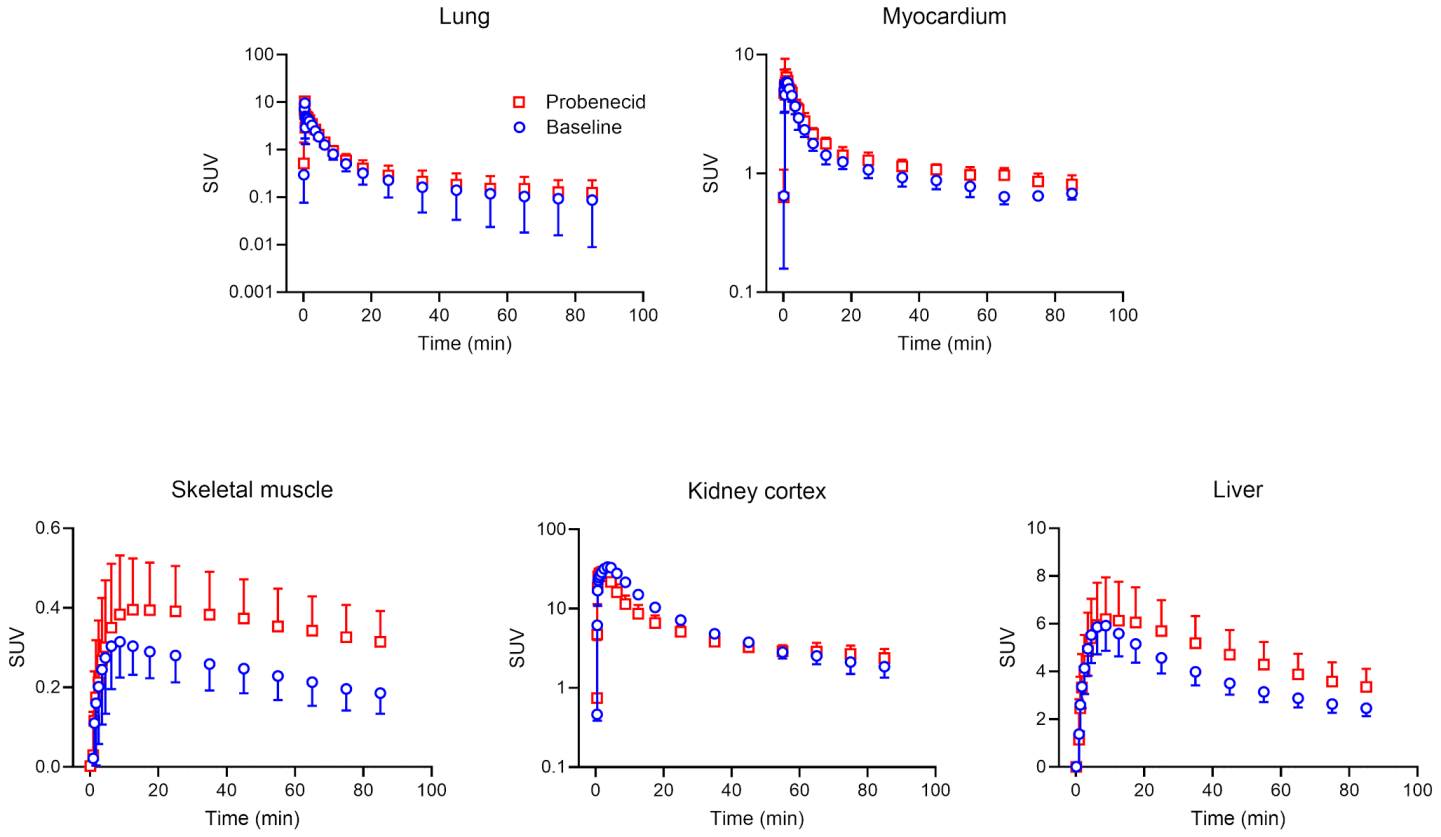
Mean (± SD) time-activity curves in peripheral MRP-expressing tissues, i.e., lung, myocardium, skeletal muscle, kidney cortex, and liver, for baseline scans and scans after probenecid administration (*n* = 7). TACs are corrected for the vascular content of radioactivity

**Fig. 7:**
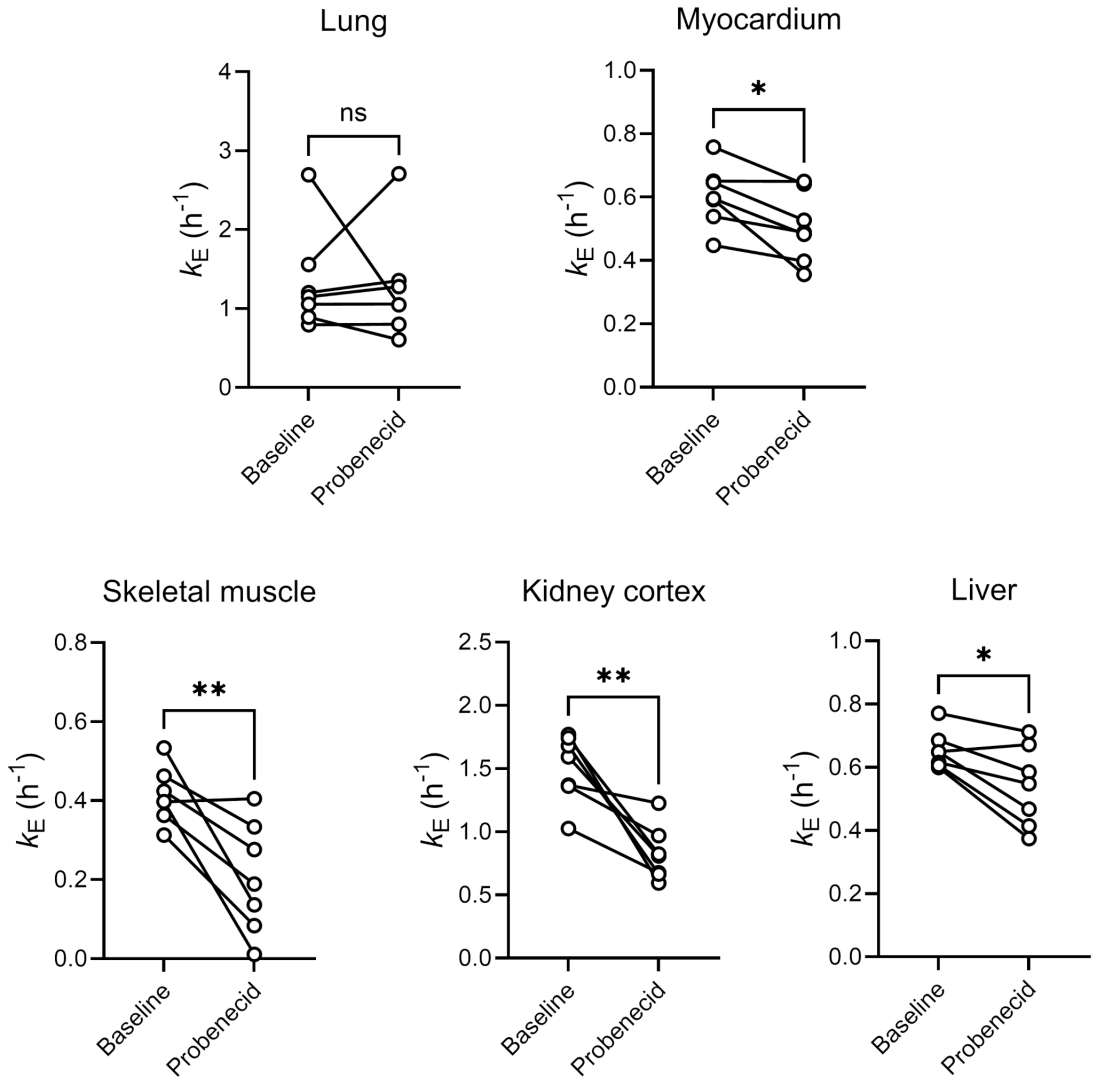
Rate constant for radioactivity elimination (*k*_E_, h^− 1^) from peripheral MRP-expressing tissues, i.e., lung, myocardium, skeletal muscle, kidney cortex, and liver, in individual subjects for baseline scans and scans after probenecid administration (*n* = 7). ns, not significant; *, *p* ≤ 0.05; **, *p* ≤ 0.01; two-sided, paired t-test

TACs for peripheral tissues (i.e., lungs, myocardium, skeletal muscle, kidney cortex, and liver) are shown in Fig. 6 and the corresponding *k*_E_ values before and after probenecid administration in Fig. 7. In the myocardium, skeletal muscle, kidney cortex, and liver significant *k*_E_ decreases occurred after probenecid administration, which exceeded TRTV values (Fig. 7, Supplementary Table 1).

## Discussion

We found significantly decreased elimination of [^11^C]BMP-derived radioactivity from several MRP-expressing tissues following probenecid administration, supporting the suitability of [^11^C]BMP to measure MRP function in humans with LAFOV PET/CT at a whole-body, multi-tissue level.

In previous rodent studies with [^11^C]BMP, *k*_E_ has been proposed as a parameter for tissue MRP1 function, which can be directly derived from the tissue TACs without the need to consider plasma radioactivity [10–12]. *k*_E_ values in the brain and lungs of *Abcc1*^*(−/−)*^ mice or MRP1 inhibitor-treated wild-type mice were markedly decreased relative to untreated wild-type mice, supporting that *k*_E_ can be used to estimate tissue MRP1 function [10–13]. The underlying assumption is that after i.v. injection [^11^C]BMP is efficiently delivered to tissues by passive diffusion followed by rapid and quantitative conversion *via* intracellular glutathione-*S*-transferases (GSTs) into its glutathione-conjugate [^11^C]MPG, which cannot diffuse back into blood due its high polarity and whose tissue elimination is dependent on MRP1 function [10]. At the same time, [^11^C]BMP is also rapidly converted in blood into [^11^C]MPG, which can only poorly distribute from blood into tissues. Therefore, after initial tissue delivery no exchange of [^11^C]BMP between blood and tissue occurs, so that the *k*_E_ parameter is assumed to reflect tissue transporter function. This has been validated in rodent studies, in which chromatographic analysis of tissue samples (i.e., brain and lung) revealed rapid and almost quantitative conversion of [^11^C]BMP into [^11^C]MPG within 5–15 min after radiotracer injection [10–12, 14]. Moreover, after i.v. injection of radiolabelled MPG into mice there was negligible brain and lung uptake of radioactivity, supporting that the glutathione-conjugate poorly distributes from blood into tissues [11, 15]. To estimate the extent of [^11^C]BMP conversion into [^11^C]MPG in human tissues, we analysed venous plasma samples, obtained at different time points after radiotracer injection, with radio-HPLC. Indeed, the major radiolabelled species in plasma represented the radiolabelled glutathione-conjugate (66 ± 5% of total radioactivity at 5 min after radiotracer injection) (Supplementary Fig. 1). We also detected some unconverted [^11^C]BMP in plasma. This may represent the plasma protein-bound fraction of [^11^C]BMP which is not available for distribution into red blood cells where glutathione-conjugation presumably occurs [16]. Moreover, an unidentified, lipophilic radiolabelled metabolite was detected in plasma (Supplementary Fig. 1), from which it is not known whether it can distribute to different tissues. Given the rapid conversion of [^11^C]BMP into [^11^C]MPG in human blood, it can be expected that efficient conversion will also occur in different human tissues in which GSTs are ubiquitously expressed, suggesting that *k*_E_ can be used in humans, like in rodents, as a parameter for tissue MRP function. To eliminate the bias of changes in blood radioactivity concentrations following probenecid administration in the calculation of *k*_E_ values, we corrected the tissue TACs for vascular radioactivity content by using tissue blood volume fractions available from literature. Since probenecid does not have any vasodilatation or vasoconstriction effects, it was assumed that tissue blood volume fractions remained unchanged after probenecid administration.

To prove transport of [^11^C]BMP-derived radioactivity by MRP1 and possibly other MRP subtypes in human tissues, study participants underwent [^11^C]BMP PET scans without and with pre-treatment with an MRP inhibitor. Since MK571 which had been previously used in rodent studies [11–14] is not available for clinical application, we instead administered the prototypical organic anion transporter inhibitor probenecid, a drug prescribed for the treatment of gout and hyperuricemia. Among other organic anion transporters (organic anion transporter 1 and 3 - OAT1/SLC22A6 and OAT3/SLC22A8 -, and MRP2-5/ABCC2-5), probenecid also inhibits MRP1 in vitro [25], and in vivo in mice [26] and humans [27]. The probenecid dose employed in our study was four times higher than the clinically used dose for the treatment of hyperuricemia. In a previous study [28], a maximum unbound plasma concentration of approximately 50 µmol/L was reached at 3–4 h after oral administration of 2 g probenecid to healthy subjects. In this concentration range, MRP1 inhibition by probenecid has been observed in vitro [25].

Following probenecid administration, the elimination of [^11^C]BMP-derived radioactivity from plasma and its urinary excretion were significantly decreased (Figs. 1b and 3a), which pointed to an inhibition of renal transporters. The decreased urinary excretion of radioactivity after probenecid administration led to increased blood radioactivity concentrations necessitating the correction of tissue TACs for vascular radioactivity content for the calculation of *k*_E_ values.

In human kidney proximal tubule cells, OAT1 and OAT3 are expressed in the basolateral (blood-facing) plasma membrane, while MRP2 and MRP4 are expressed in the apical brush-border membrane (Supplementary Fig. 5d) [3]. These transporters work together in mediating the urinary excretion of various drugs and drug metabolites [3]. Experiments in *Abcc4*^*(−/−)*^ mice had shown that MRP4 contributes to the urinary excretion of [^11^C]BMP-derived radioactivity in mice [12]. Our observation that CL_urine, plasma, total_ was reduced to a greater extent than CL_urine, kidney, total_ (Fig. 3b) suggests that both basolateral uptake transporters (i.e., OAT3) and apical efflux transporters (MRP2, MRP4) are involved in the urinary excretion of [^11^C]BMP-derived radioactivity (Supplementary Fig. 5d). This is in good agreement with mouse experiments, in which MK571 inhibited both the renal uptake and efflux clearances of [^11^C]BMP-derived radioactivity [12]. The decrease in CL_urine, plasma, [_^11^_C]MPG_ was greater than that of CL_urine, kidney, total_ (Fig. 3b) indicating that [^11^C]MPG is the radiolabelled species which is preferentially excreted *via* renal organic anion transporters. In addition to the decreased urinary clearance values, *k*_E_ was also significantly reduced in the kidneys (Fig. 7).

We also observed a significant *k*_E_ decrease in the liver (Fig. 7). However, the amount of radioactivity excreted into bile was low (< 5% of the administered activity) and not affected by probenecid administration (Supplementary Fig. 3). The *k*_E_ reduction in the liver may therefore not be due to inhibition of MRP2-mediated biliary excretion of radioactivity, but rather to inhibition of MRP3- and/or MRP4-mediated efflux of radioactivity from hepatocytes into blood (Supplementary Fig. 5e) [3].

Apart from the effects seen in excretory organs, we observed significant *k*_E_ decreases in the myocardium and skeletal muscle after probenecid administration (Fig. 7). MRP1 is expressed in the sarcolemmal membrane of cardiomyocytes [29] and skeletal muscle fibres [30] (Supplementary Fig. 5b, c). In the myocardium, MRP1 was shown to protect the heart from doxorubicin-induced cardiotoxicity by direct cellular export of doxorubicin and/or by cellular export of cytotoxic products generated during doxorubicin-induced oxidative stress [29]. In skeletal muscle, MRP1 was shown to control intracellular concentration levels of lipid-lowering statin drugs and to protect muscle cells from statin-induced muscle toxicity [30].

In the lungs, MRP1 is abundantly expressed in the basolateral membrane of airway epithelial cells (Supplementary Fig. 5a) [31]. [^11^C]BMP has been successfully used to measure MRP1 function in the rodent lungs either after i.v. administration [11, 12] or after intratracheal aerosolisation [14]. However, in the present study no significant *k*_E_ decrease was observed in the lungs following probenecid administration (Fig. 7). This may be explained by insufficiently high tissue probenecid concentration levels to achieve effective inhibition of MRP1 in airway epithelial cells.

In the brain, MRP1 is predominantly expressed in parenchymal cells (i.e., neurons and astrocytes) (Supplementary Fig. 4a) and in choroid plexus epithelial cells (Supplementary Fig. 4b) and only to a low extent in brain capillary endothelial cells [32]. It has been proposed that [^11^C]BMP is converted in mouse brain parenchymal cells by GSTs into [^11^C]MPG, which is exported from cells by MRP1 and eliminated across the mouse BBB by OAT3 and MRP4 [15]. As opposed to the mouse brain (*k*_E_: 1.5 ± 0.06 h^− 1^) [10, 12], [^11^C]BMP-derived radioactivity was very slowly eliminated from the human brain, pointing to substantial species differences in the expression of OAT3, MRP4 and possibly other transporters at the mouse and human BBB [16]. This is supported by quantitative proteomics data, which revealed that OAT3 expression was below the limit of detection in human brain capillary endothelial cells [33]. *k*_E_ values were 50–90 times lower in the human than in the mouse brain and neither of the two analysed brain regions (cortex and cerebellum) showed significant *k*_E_ changes following probenecid administration (Fig. 5). This contrasts with mouse data, which show a significant *k*_E_ decrease in the brain after administration of the non-subtype-specific MRP inhibitor MK571 [12, 13]. While it is not certain whether probenecid crossed the BBB in sufficient amounts to inhibit MRP1 in brain parenchymal cells at the administered dose, the slow brain washout of radioactivity suggests that [^11^C]BMP may not be suitable to measure MRP1 function in the human brain.

Surprisingly, we observed a distinct regional radioactivity uptake pattern in the brain with preferential grey matter accumulation and higher accumulation in cerebellar as compared with cortical grey matter (Fig. 2b). This cerebral uptake pattern closely resembles that of the SPECT tracer [^99m^Tc]Tc-meso-hexamethyl propyleneamine oxime (HMPAO), which is a stereoisomer of the clinically used perfusion imaging tracer [^99m^Tc]Tc-d, l-HMPAO [34]. It has been proposed that [^99m^Tc]Tc-meso-HMPAO crosses the BBB by passive diffusion and is inside the brain converted into a hydrophilic glutathione-conjugate which is retained in brain tissue. As the rate-limiting step in the brain kinetics of [^99m^Tc]Tc-meso-HMPAO is its conversion into the glutathione-conjugate, its regional brain uptake depends on regional glutathione content. Experiments in mice revealed preferential grey matter accumulation of [^99m^Tc]Tc-meso-HMPAO-derived radioactivity, higher accumulation in the cerebellum than in the cortex, and a good correlation between regional brain uptake and regional glutathione concentration [34]. Given that [^11^C]BMP also undergoes glutathione-conjugation and is trapped in the human brain, it is tempting to speculate that the distinct regional uptake pattern of [^11^C]BMP-derived radioactivity may be related to regional differences in the glutathione-conjugation system (i.e., glutathione concentration and/or GST activity). Since glutathione content is an important marker of oxidative stress and since no PET tracer for mapping the glutathione-conjugation system in the brain is currently available, further investigations including mechanistic preclinical studies are warranted to assess the suitability of [^11^C]BMP for this application.

Apart from brain parenchymal cells, MRP1 is expressed in the basolateral (blood-facing) membrane of choroid plexus epithelial cells (Supplementary Fig. 4b), where it restricts the distribution of its substrates from blood into cerebrospinal fluid [35]. Choroid plexus epithelial cells possess high GST activity, which forms together with MRP1 an efficient cellular detoxification system [36]. As opposed to the slow brain kinetics of radioactivity, the choroid plexus showed appreciable washout of radioactivity suggesting the presence of some efflux mechanism (Fig. 4). Indeed, radioactivity concentrations in the ventricular system were 3–4 times lower than in brain tissue, which suggested restricted distribution of radioactivity across the blood-cerebrospinal fluid barrier. However, similar to the brain, probenecid administration had no effect on *k*_E_ in the choroid plexus (Fig. 5). Since blood capillaries of the choroid plexus are fenestrated, it appears unlikely that insufficient probenecid exposure was the cause for the lack of an effect of probenecid on the kinetics of [^11^C]BMP-derived radioactivity in the choroid plexus. In contrast, we observed a pronounced *k*_E_ reduction in the retina (Fig. 5), in which MRP1 is expressed in the plasma membrane of the retinal pigment epithelium forming the outer blood-retina barrier (Supplementary Fig. 4c) [37]. Even though we were not able to correct the retinal TACs for vascular radioactivity content, it appears unlikely that the effect seen in the retina was only caused by a blood effect, as the *k*_E_ reduction in the retina (− 57 ± 29%) largely exceeded the *k*_E_ reduction in blood (− 26 ± 10%). The exact reasons for the discrepancy between the effect of probenecid on radioactivity kinetics in the choroid plexus and retina remain unclear.

Even though BMP is not a clinically used drug, it can be considered as a drug-like molecule which has similar physicochemical properties as many small-molecule drugs. Transporter-mediated DDIs are of great concern in drug development and regulatory authorities currently mandate to assess the DDI risk for new drug candidates [3]. When a DDI risk cannot be excluded based on available in vitro data, clinical studies are required. In most clinical DDI studies, drug concentrations are only assessed in plasma and changes in tissue pharmacokinetics, which could for instance cause organ toxicity (e.g., statin-induced rhabdomyolysis), may therefore remain undetected [5]. PET employing microdoses of radiolabelled drugs can be used to safely assess DDIs in humans at a tissue level [5]. Up to now, this has only been possible in single organs due to the limited axial FOV of previously available clinical PET scanners [38]. The availability of LAFOV PET scanners can thus be considered as a major step forward, as it will allow for assessing drug disposition and DDIs in humans at a whole-body, multi-tissue level. In this context, unique advantages of LAFOV PET scanners include the possibility to obtain an image-derived blood input function from a large blood vessel within the FOV of the scanner (i.e., the aorta) and their high sensitivity which potentially allows for longer dynamic scan durations than with conventional PET scanners, thus enabling improved pharmacokinetic analysis.

## Conclusion

While [^11^C]BMP appears unsuitable to measure MRP1 function in the human brain, it may be used to measure the function of MRP1 and possibly other MRP subtypes in peripheral tissues employing *k*_E_ as a parameter for tissue transporter function that can be directly derived from the blood-corrected tissue TACs. This could be applied for studying the role of these transporters in different diseases and for assessing transporter-mediated DDIs at a whole-body level. Future work needs to assess the MRP subtype specificity of [^11^C]MPG to elucidate which MRP subtypes other than MRP1 were involved in its peripheral disposition.

## Electronic supplementary material

Below is the link to the electronic supplementary material.

Supplementary Material 1

## Data Availability

The datasets generated during and analysed during the current study are available from the corresponding author on reasonable request (OL).
