# Supplementary material for: Effect of probenecid on the whole-body disposition of 6-bromo-7-[11C]methylpurine in humans assessed with long axial field-of-view PET/CT
Source: Eur J Nucl Med Mol Imaging. 2025 Feb 8;52(7):2477–90. doi: 10.1007/s00259-025-07121-5 (PMC12119383; doi:10.1007/s00259-025-07121-5)
Supplement: Supplementary file 1 — Supplementary Material 1 [file 259_2025_7121_MOESM1_ESM.docx]

**Supplementary Information**

**Effect of probenecid on the whole-body disposition of 6-bromo-7-[^11^C]methylpurine in humans assessed with long axial field-of-view PET/CT**

Matthias Jackwerth^1^, Severin Mairinger^1,2^, Ivo Rausch^3^, Maria Weber^1^, Anselm Jorda^1^, Lukas Nics^2^, Werner Langsteger^2^, Markus Zeitlinger^1^, Marcus Hacker^2^, Oliver Langer^1,2^

^1^Department of Clinical Pharmacology, Medical University of Vienna, Vienna, Austria

^2^Department of Biomedical Imaging and Image-guided Therapy, Medical University of Vienna, Vienna, Austria

^3^QIMP Team, Center for Medical Physics and Biomedical Engineering, Medical University of Vienna, Vienna, Austria

**Supplementary Table 1** *k*_E_ values in different tissues for baseline scans and scans after probenecid administration (*n* = 7 each)

| **Tissue** ^a^ | ***k*_E_ (h^−1^)**  **Baseline** | ***k*_E_ (h^−1^)**  **Probenecid** | **% Change** | **TRTV (%)** ^b^ |
| --- | --- | --- | --- | --- |
| Cortex | 0.028 ± 0.012 | 0.034 ± 0.012 | 34 ± 57 | −12 ± 41 |
| Cerebellum | 0.017 ± 0.010 | 0.019 ± 0.014 | 22 ± 53 | 0.4 ± 83 |
| Choroid plexus | 0.283 ± 0.037 | 0.273 ± 0.062 | −3 ± 20 | 0.1 ± 16 |
| Retina | 0.213 ± 0.045 | 0.094 ± 0.059 ^c^ | −57 ± 29 | 30 ± 38 |
| Lung | 1.335 ± 0.648 | 1.265 ± 0.687 | 1 ± 42 | −30 ± 50 |
| Myocardium | 0.604 ± 0.097 | 0.506 ± 0.111 ^d^ | −16 ± 12 | 10 ± 28 |
| Skeletal muscle | 0.413 ± 0.071 | 0.205 ± 0.141 ^c^ | −51 ± 34 | 2 ± 21 |
| Kidney cortex | 1.506 ± 0.269 | 0.823 ± 0.217 ^c^ | −43 ± 20 | 14 ± 16 |
| Liver | 0.654 ± 0.060 | 0.539 ± 0.127 ^d^ | −18 ± 15 | 6 ± 10 |

Values are reported as mean ± standard deviation

^a^ Tissue time-activity curves were corrected for the vascular contribution of radioactivity except for the choroid plexus and retina

^b^ Test-retest variability (%) of *k*_E_ was re-calculated from test-retest scans in 6 subjects (see reference [15], main manuscript) using blood-corrected tissue time-activity curves (except for the choroid plexus and retina)

^c^ Significantly different from baseline scan (*p* ≤ 0.01, two-sided, paired t-test)

^d^  Significantly different from baseline scan (*p* ≤ 0.05, two-sided, paired t-test)

**
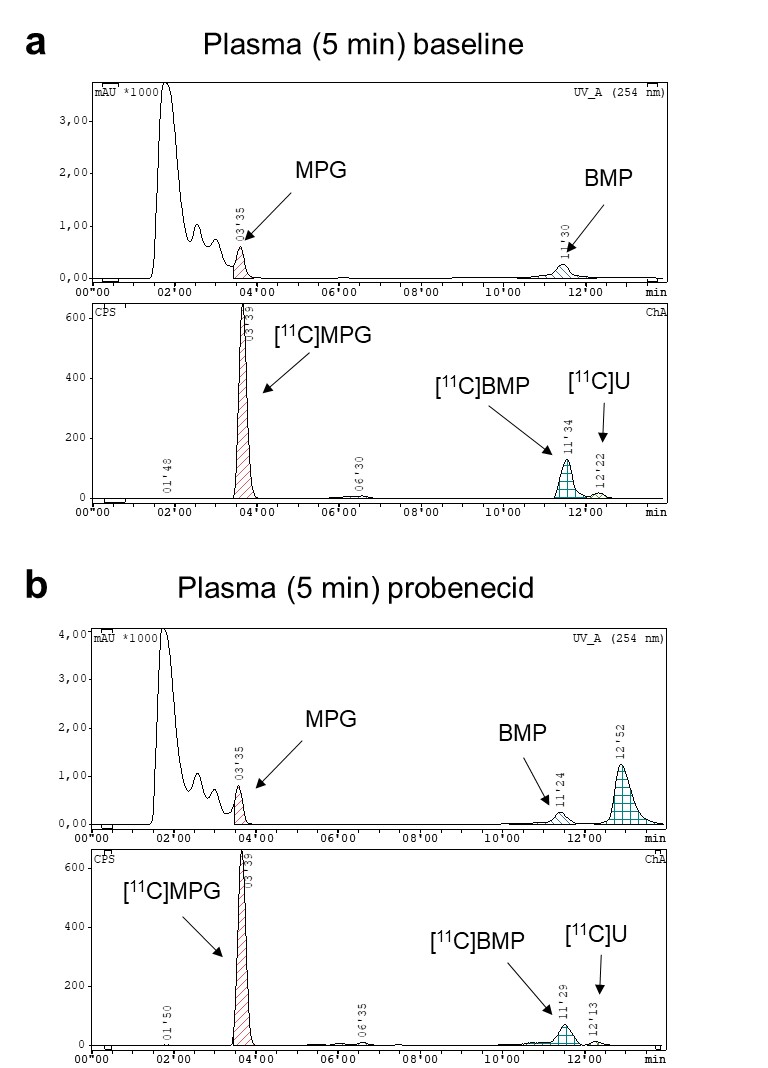
**

**Supplementary Fig. 1** Representative radio-HPLC chromatograms for analysis of plasma collected in one subject at 5 min after [^11^C]BMP injection for the baseline scan (**a**) and the scan after probenecid administration (**b**). The upper channel represents UV absorption (254 nm) and the lower channel radioactivity detection. Samples were spiked with unlabelled BMP and MPG. [^11^C]U is an unidentified, lipophilic radiolabelled metabolite

**
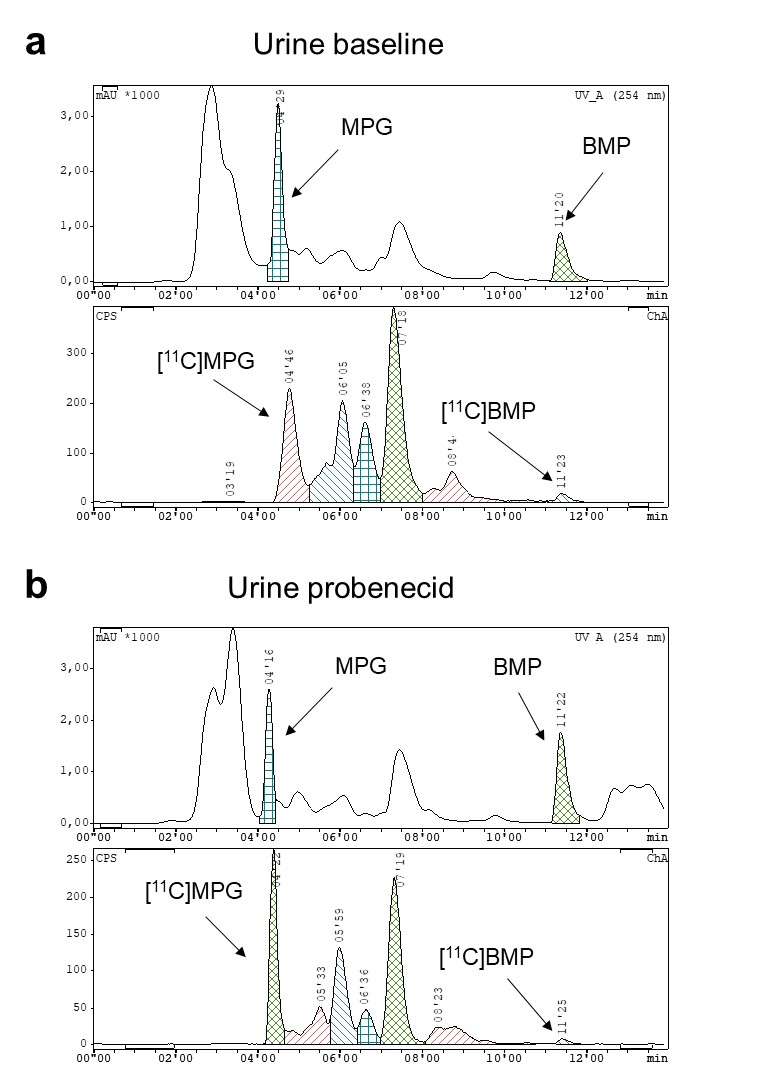
**

**Supplementary Fig. 2** Representative radio-HPLC chromatograms for analysis of urine collected in one subject at the end of the baseline scan (**a**) and probenecid scan (**b**). The upper channel represents UV absorption (254 nm) and the lower channel radioactivity detection. Samples were spiked with unlabelled BMP and MPG


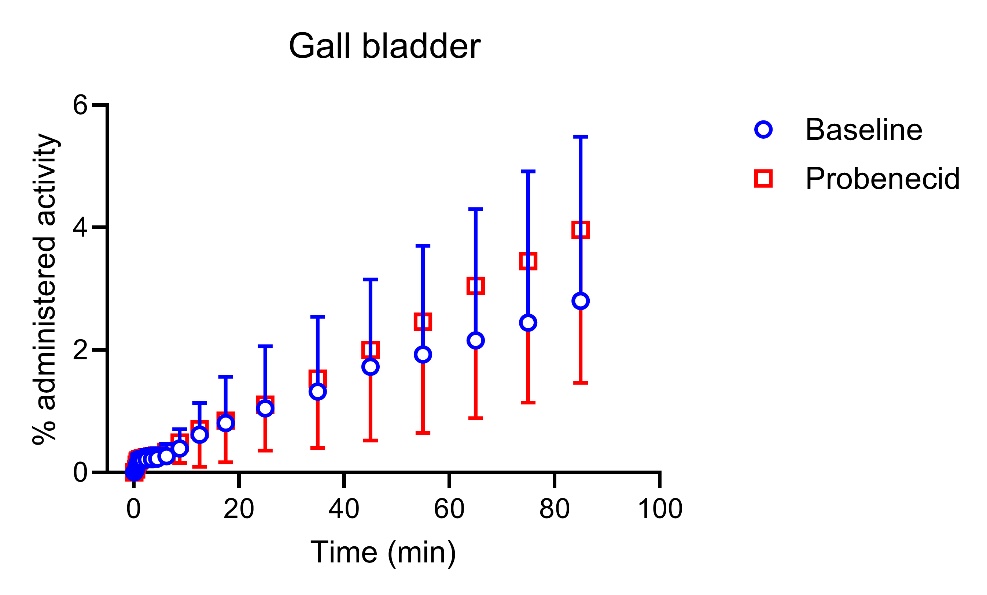


**Supplementary Fig. 3** Mean (± SD) time-activity curves for the excretion of [^11^C]BMP-derived radioactivity into the gall bladder for baseline scans and scans after probenecid administration (*n* = 7)

**a**

**b**

**c**


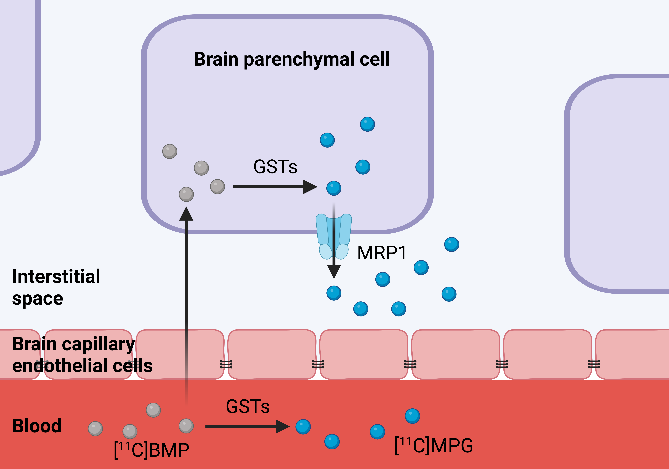

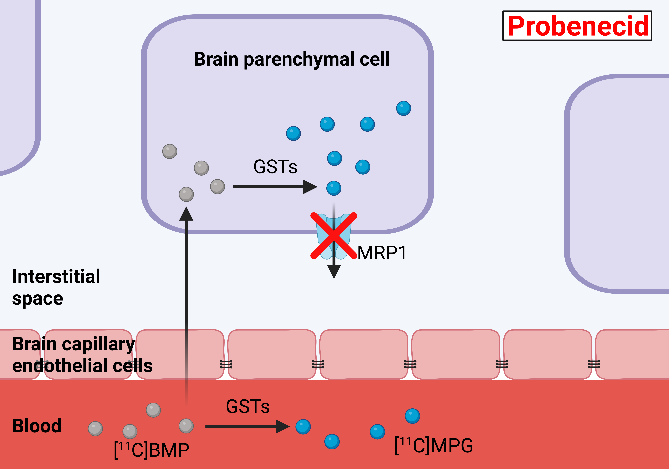

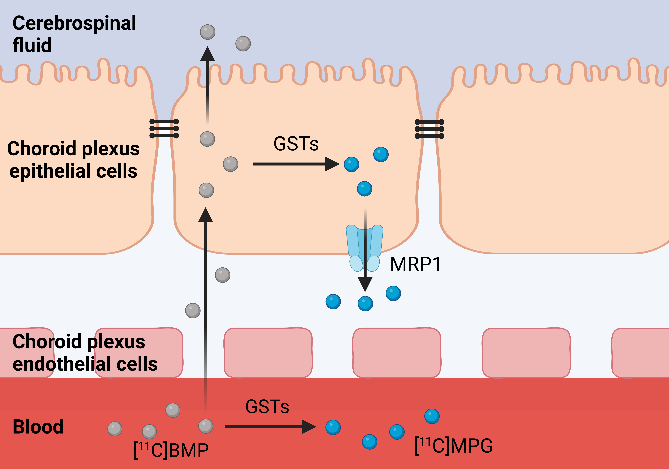

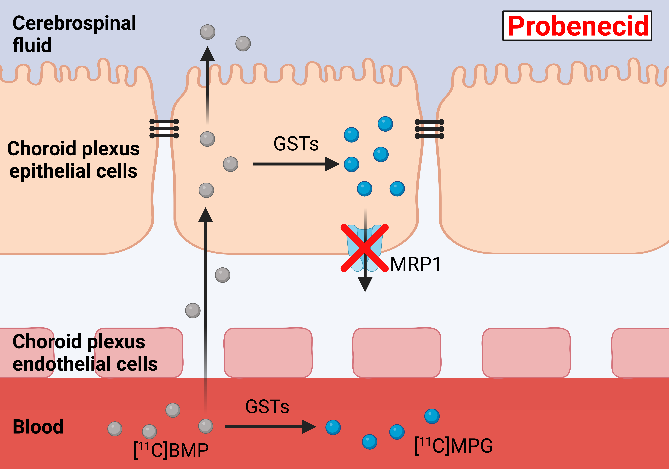

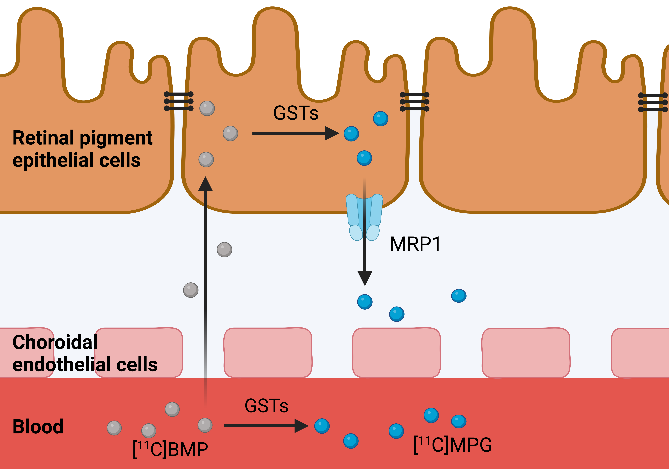

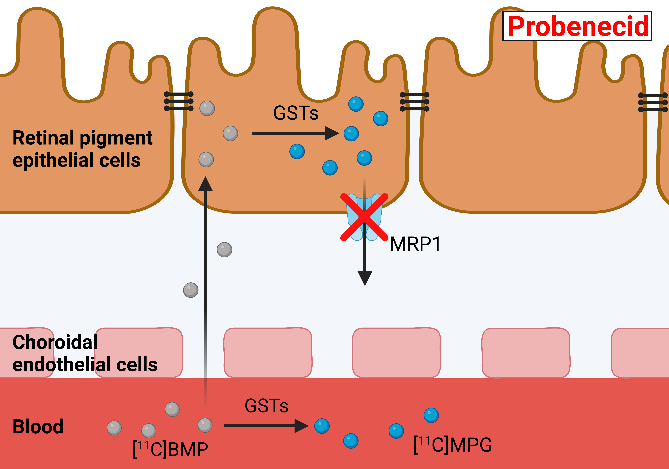


**Supplementary Fig. 4** Illustration of the localisation of MRP1 in the analysed central tissues (**a**: brain, **b**: choroid plexus, and **c**: retina) and the expected effect of transporter inhibition with probenecid on the disposition of [^11^C]BMP-derived radioactivity. The figure was created with BioRender.com

**a**

**b**

**c**

**d**


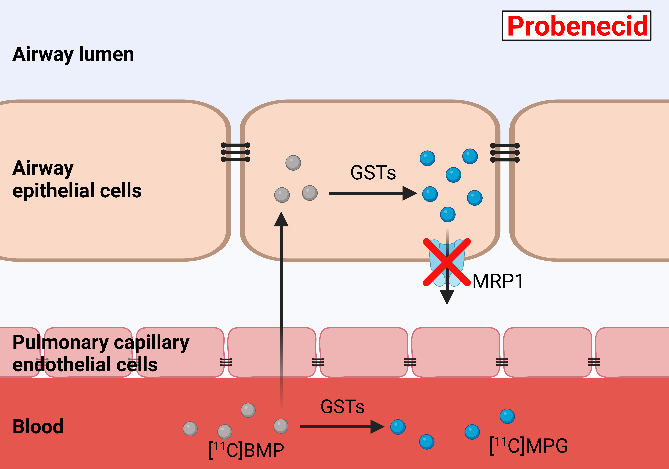

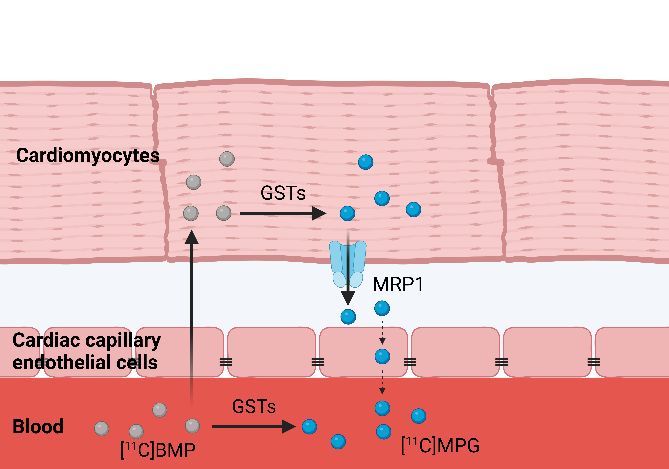

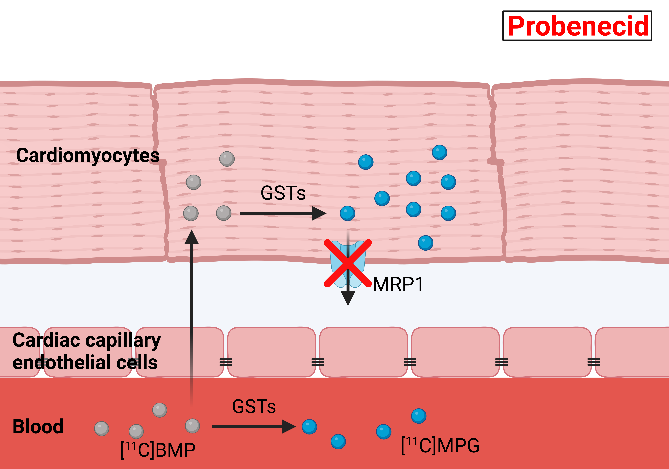

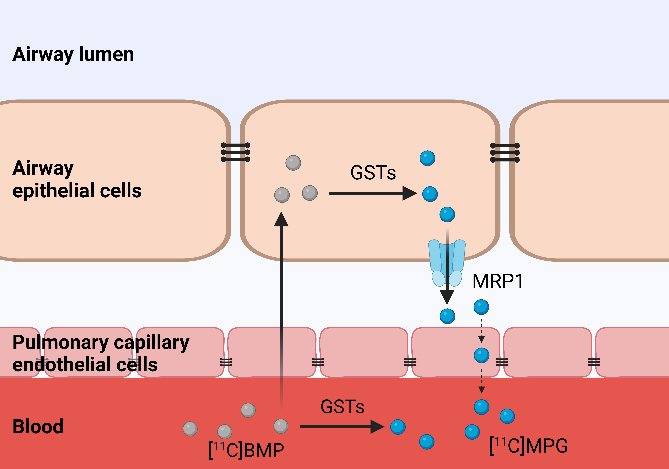

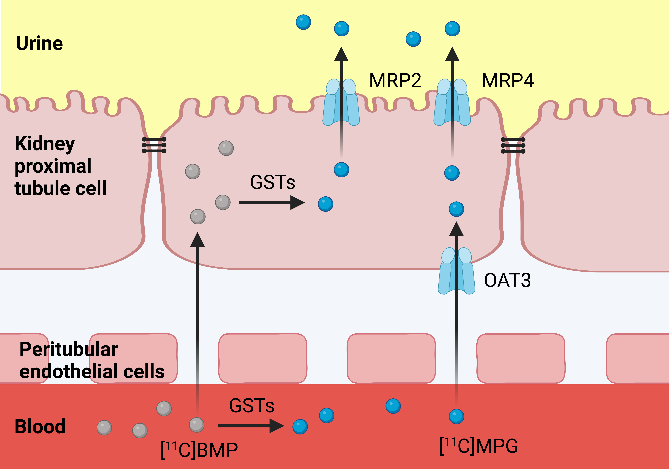

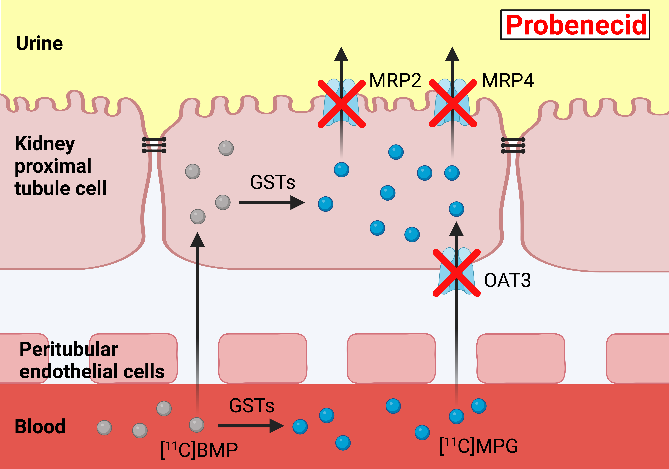

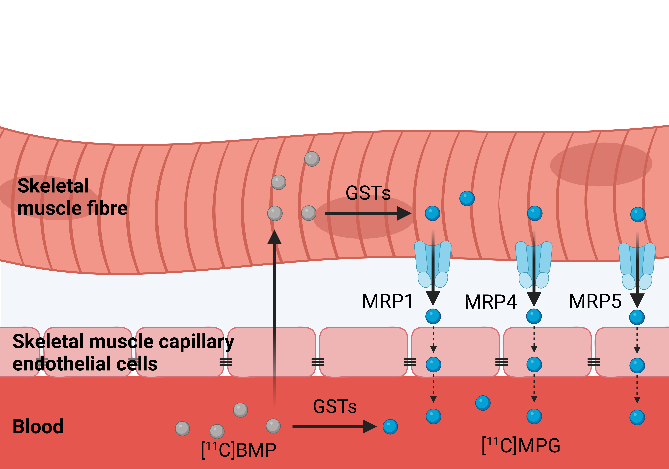

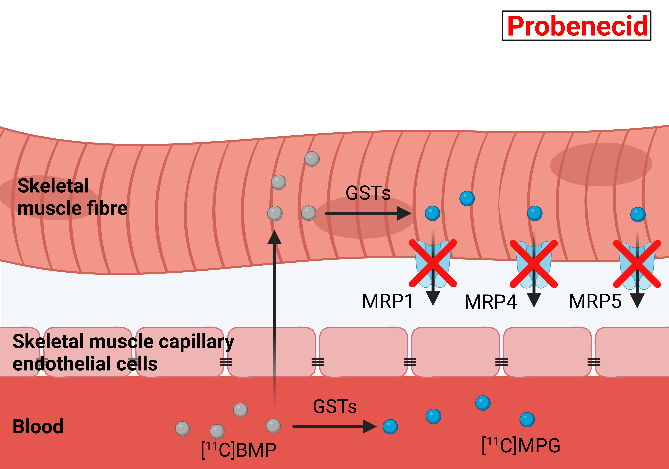


**e**


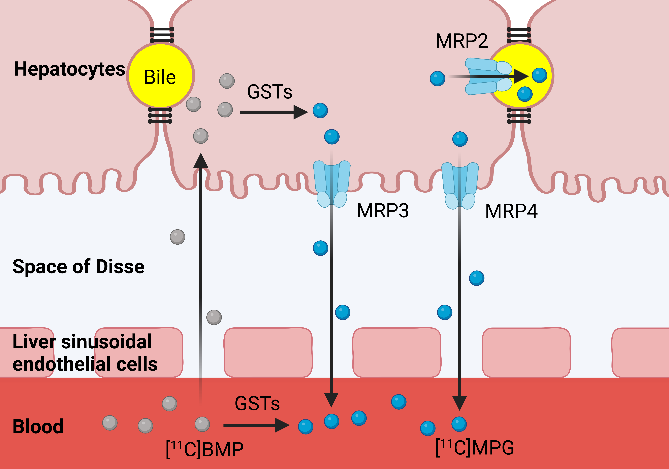

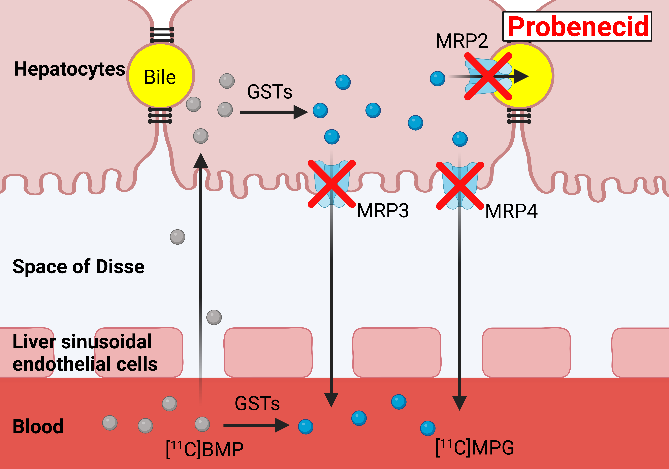


**Supplementary Fig. 5** Illustration of the localisation of MRP1 and other MRP subtypes in the analysed peripheral tissues (**a**: lungs, **b**: myocardium, **c**: skeletal muscle, **d**: kidney cortex, and **e**: liver) and the expected effect of transporter inhibition with probenecid on the disposition of [^11^C]BMP-derived radioactivity. Dotted arrows indicate unknown mechanisms of membrane passage. The figure was created with BioRender.com
